# Probing conformational changes of monomeric transthyretin with second derivative fluorescence

**DOI:** 10.1038/s41598-019-47230-4

**Published:** 2019-07-29

**Authors:** Denisa Jazaj, Seyyed Abolghasem Ghadami, Francesco Bemporad, Fabrizio Chiti

**Affiliations:** Dipartimento di Scienze Biomediche Sperimentali e Cliniche “Mario Serio”, Sezione di Scienze Biochimiche, Università degli Studi di Firenze, Viale Morgagni 50, 50134 Firenze, Italy

**Keywords:** Biophysics, Proteins, Optical spectroscopy

## Abstract

We have studied the intrinsic fluorescence spectra of a monomeric variant of human transthyretin (M-TTR), a protein involved in the transport of the thyroid hormone and retinol and associated with various forms of amyloidosis, extending our analysis to the second order derivative of the spectra. This procedure allowed to identify three peaks readily assigned to Trp41, as the three peaks were also visible in a mutant lacking the other tryptophan (Trp79) and had similar FRET efficiency values with an acceptor molecule positioned at position 10. The wavelength values of the three peaks and their susceptibility to acrylamide quenching revealed that the three corresponding conformers experience different solvent-exposure, polarity of the environment and flexibility. We could monitor the three peaks individually in urea-unfolding and pH-unfolding curves. This revealed changes in the distribution of the corresponding conformers, indicating conformational changes and alterations of the dynamics of the microenvironment that surrounds the associated tryptophan residue in such transitions, but also native-like conformers of such residues in unfolded states. We also found that the amyloidogenic state adopted by M-TTR at mildly low pH has a structural and dynamical microenvironment surrounding Trp41 indistinguishable from that of the fully folded and soluble state at neutral pH.

## Introduction

Human transthyretin (TTR) is a protein consisting of a tetramer of four identical subunits^1,2^. It is predominantly synthesized by liver hepatocytes, epithelial cells of the brain choroid plexus, and the retinal pigment epithelium, but also in many other tissues to a lower extent^1,2^. In the plasma, TTR is a secondary transporter of the thyroid hormone and a primary transporter of the retinol binding protein (RBP), whereas in the cerebrospinal fluid (CSF) it is the main transporter of the thyroid hormone^1,3,4^.

The misfolding and aggregation of TTR to form different types of fibrillar aggregates, known as amyloid fibrils, is associated with several human pathologies, collectively referred to as ATTR^5,6^. In these diseases, the aggregates have a well-defined fibrillar appearance, a cross-β structure and characteristic tinctorial properties following the staining with amyloid-diagnostic dyes^7,8^. Deposition of amyloid by wild-type TTR in the heart occurs for 10–25% of humans older than 80, resulting in a pathological condition known as senile systemic amyloidosis (SSA)^9,10^. Amyloid formation by any of the ca. 100 different variants of TTR so far described leads to early-onset TTR amyloidosis with autosomal dominant inheritance, such as familial amyloid cardiomyopathy (FAC), familial amyloid polyneuropathy (FAP), and leptomeningeal amyloidosis^11,12^. Hence, the predominant species within amyloid fibrils in TTR-related familial conditions is mutant TTR^7,13–15^. The single mutations that give rise to such conditions do not significantly perturb the structure of tetrameric TTR, but rather reduce the kinetic and/or thermodynamic stability of native TTR in its tetrameric form, so that the population of the partially unfolded amyloidogenic intermediate in the monomeric form is increased^5,16,17^.

The TTR community has expended considerable effort to investigate the structure and dynamics of potentially relevant forms of TTR, including wild-type tetrameric TTR^18–22^, a monomeric conformational state populated at low pH or following a specific double mutation^19,20,23–26^ and amyloid fibrils^27,28^. Such efforts have been made not just under physiological conditions, but also under a number of experimental conditions known to promote amyloid fibril formation by stabilising partially folded monomeric states, possibly acting as amyloidogenic precursor states; these include mildly acidic pH values^19,20,24,26^, high values of hydrostatic pressure^29^, small concentrations of urea^26,30^ or native conditions after a jump of urea concentration to detect partially folded states accumulating during folding^26,30^.

The intrinsic fluorescence of tryptophan residues contained in proteins has been widely used to determine and monitor the structural changes occurring during protein conformational conversions. Advantages of this technique over other methods include its high sensitivity, the small protein contents and concentrations required to obtain high quality fluorescence spectra, the high signal-to-noise ratio, the rapidity of data acquisition, the sensitivity of the wavelength of maximum fluorescence (λ_max_) and quantum yield to even subtle structural changes^31–34^. Nevertheless, intrinsic tryptophan fluorescence has many additional potentialities that have not been fully exploited to monitor protein conformational changes. One of these is the analysis of the second derivative of fluorescence spectra to identify contributions of different tryptophan residues, or even different conformations of individual tryptophan residues, to the overall fluorescence spectra^35,36^. Such an approach has been used to study a few proteins in the past^35–39^, but has never been applied to study any TTR form in any of its conformational states, and has not reached any substantial level of detail even in other protein systems.

Monomeric TTR has two tryptophan residues, numbered 41 and 79. Trp41 has a very important location, because it is in β-strand C that has been described to be either fully folded or unfolded in the amyloidogenic state populated at acidic pH in the different structural studies reported so far^19–22,24,26^. In this manuscript we have thus analysed the gross fluorescence spectra and their second derivative of the F87M/L110M mutant of TTR that has been designed to be stable as a monomer (M-TTR)^23^, and of another mutant carrying the additional single-point W79F substitution, thus containing the single tryptophan at position 41. We have carried out this analysis under a variety of conditions where fully folded, fully unfolded or partially folded (and potentially amyloidogenic states) are populated. We will show that it is possible to monitor various conformations of individual tryptophan residues during the various structural transitions involving the whole protein, which allows determining not just changes on the chemical environment around the tryptophan residues, but also modifications of their dynamics as the overall structural transitions occur and native-like conformers of such residues.

## Results

### M-TTR and its W79F mutant have complex intrinsic fluorescence spectra

**Figure 1:**
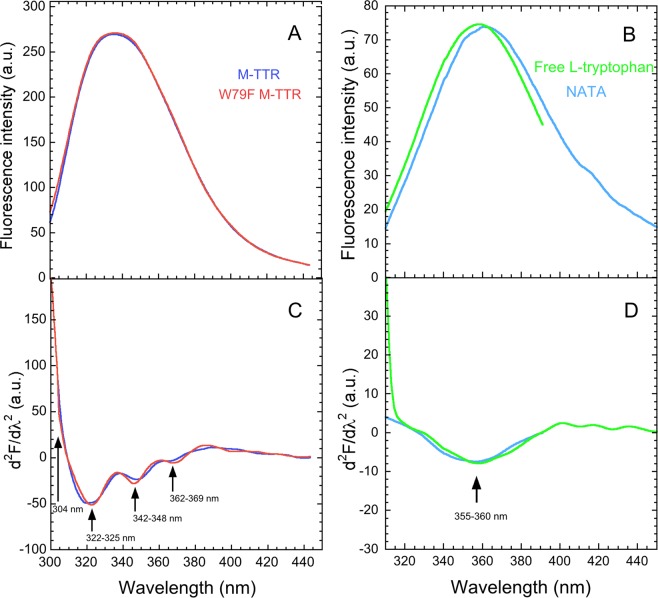
Gross and second derivative fluorescence spectra of M-TTR, W79F M-TTR, free L-tryptophan and NATA. (**A**,**B**) Fluorescence emission spectra (excitation 290 nm, slits 5 and 10 nm for ex and em, respectively) of M-TTR and its W79F mutant (**A**) and of free L-tryptophan and NATA (**B**) at a concentration of 3 µM, in 20 mM phosphate buffer, pH 7.4, 25 °C. (**C**,**D**) Calculated second derivative spectra of M-TTR and W79F M-TTR (**C**) and of free L-tryptophan and NATA (**D**).

We initially recorded the intrinsic fluorescence spectra (290 nm excitation) of M-TTR and W79F M-TTR in 20 mM phosphate buffer, pH 7.4, 25 °C at a protein concentration of 3 µM (Fig. 1A). The two spectra were found to be superimposable and very similar in terms of fluorescence intensity, indicating that only Trp41 contributes to the overall intrinsic fluorescence of folded M-TTR and that the fluorescence of Trp79 is quenched in this conformation. This was previously observed for tetrameric WT-TTR^19^ and confirmed for M-TTR under our conditions of analysis (Fig. 1A).

In mathematical terms, a fluorescence emission spectrum represents the fluorescence emission intensity (F, arbitrary units) at various values of wavelength (λ, units of nm), at a given excitation wavelength, and the two spectra reported in Fig. 1A follow this well-established convention. We calculated their second derivative, which consists of the second order derivative of F with respect to λ (d^2^F/dλ^2^) and plotted them in Fig. 1C. The second derivative spectra of M-TTR and W79F M-TTR reveal three peaks at *ca*. 322–325 nm, 342–348 nm and 362–369 nm and a shoulder at ca. 304 nm (Fig. 1C). The 304 nm peak can be assigned to one or more of the five tyrosine residues of M-TTR, as 304 nm is just the wavelength of maximum fluorescence for tyrosine side chains^34^. The three remaining peaks are located in a wavelength range typical of tryptophan residues^34,40^, but the question arises as to how a single residue, such as Trp41, can give rise to three peaks with such distinct wavelengths.

The fluorescence emission spectra of free L-tryptophan and *N*-Acetyl-L-tryptophanamide (NATA), as well as their second derivative spectra, are characterised by one major peak at ca. 355–360 nm (Fig. 1B,D), as expected for these compounds^34,40^. The presence of three peaks in the fluorescence spectra of M-TTR and its W79F mutant, in the wavelength window of 320–370 nm, is therefore a peculiarity of this protein and its mutant.

### The three peaks at 320–370 nm in the second derivative spectra arise from Trp41

**Figure 2:**
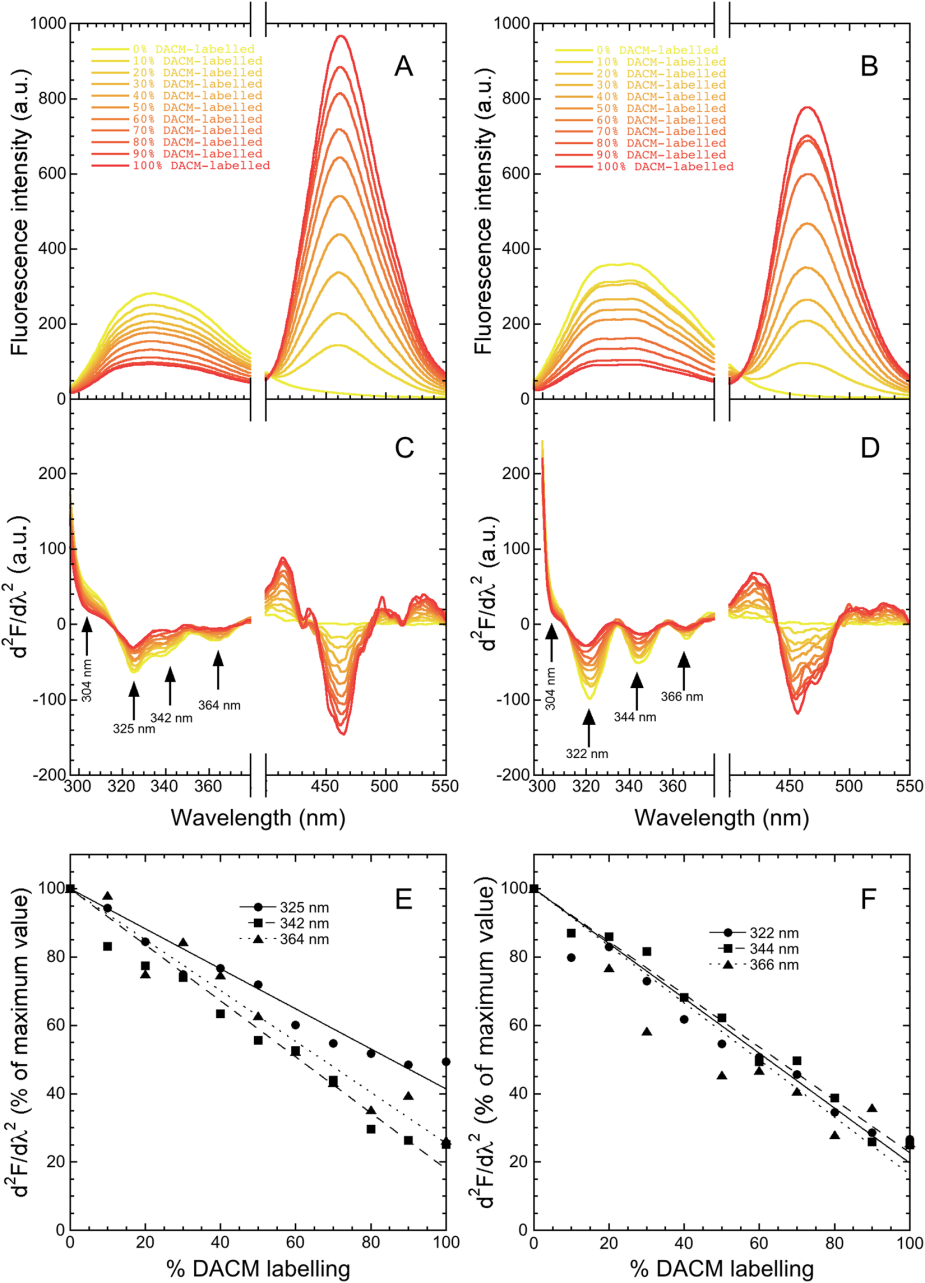
Gross and second derivative fluorescence spectra of M-TTR and W79F M-TTR with various degrees of DACM-labelling. (**A**,**B**) Fluorescence spectra (excitation 290 nm, slits 2.5 and 5 nm for ex and em, respectively) of mixtures of M-TTR and DACM-M-TTR (**A**) or W79F M-TTR and W79F DACM-M-TTR (**B**) at the indicated percentages of the DACM-labelled protein, at 3 µM total protein concentration, in 20 mM phosphate buffer, pH 7.4, 25 °C. (**C**,**D**) Corresponding second derivative spectra for M-TTR (**C**) and W79F M-TTR (**D**) with arrows indicating the major peaks arising from tyrosine residues (304 nm) and tryptophan residues (320–370 nm). (**E**,**F**) Values of second derivative as a function of DACM-labelling percentage for the three indicated peaks of M-TTR (**E**) and W79F M-TTR. (**F**) To facilitate the comparison, all values were normalised attributing 100% to those determined with 0% labelling. The lines through the data represent the best fits to a linear function of the form d^2^F/dλ^2^ = 100% + *a* · (% DACM labelling).

In order to investigate the origin of the three peaks in the second derivative fluorescence spectra of M-TTR and its W79F mutant in the wavelength region of 320–370 nm, we acquired the fluorescence spectra (290 nm excitation) of the two protein variants labelled with N-(7-dimethylamino-4-methylcoumarin-3-yl)maleimide (DACM), at Cys10 (DACM-M-TTR and W79F DACM-M-TTR) with various degrees of labelling ranging from 0% to 100% (Fig. 2A,B). DACM is a coumarin derivative acting as an acceptor of the Trp41 fluorescence in fluorescence resonance energy transfer (FRET), which therefore acts as a FRET donor^26^. All spectra were again acquired in 20 mM phosphate buffer, pH 7.4, 25 °C at a protein concentration of 3 µM. As observed previously^26^, for both protein variants the tryptophan fluorescence emission at 320–370 nm decreases with the increase of DACM labelling, indicating the presence of effective FRET (Fig. 2A,B). Concomitantly, the DACM fluorescence emission at 463 nm increases with the increase of DACM labelling, again indicating the occurrence of FRET (Fig. 2A,B).

We then calculated second derivatives of these spectra (Fig. 2C,D). The tyrosine peak at 304 nm and the three peaks at 320–370 nm are present in all cases. The three peaks at 320–370 nm, unlike the peak at 304 nm, decrease in intensity with DACM labelling in both protein variants (Fig. 1C–F), suggesting that they all arise from Trp41 fluorescence that is effectively transferred to DACM through FRET. The peak at 304 nm does not decrease and indeed increases apparently in intensity, most probably because the decrease of the other three peaks unravel it more markedly. The three peaks at 320–370 nm decrease to similar extents with DACM labelling (Fig. 1E,F). The peak at 325 nm of M-TTR is the only one that decreases less markedly relative to the other two (Fig. 1E), but this behaviour was not found for the corresponding peak in the W79F mutant (Fig. 1F), implying that the apparent change is not significant. The finding that the second derivative values of the three peaks decrease to similar extents in the presence of DACM-labelling indicates that they all arise from donors located spatially at very similar distances from the DACM acceptor, as the FRET efficiency is highly dependent on the donor-acceptor distance^26,34,41^. These observations indicate that the three peaks that are apparent in the 320–370 nm region of the second derivative spectra all arise from Trp41, possibly from three different conformers (rotamers) of this residue. The different wavelengths of emitted fluorescence of the three Trp41 conformers suggest that they have different degrees of hydrophobic contacts, hydrogen bonding and solvent exposure in the native protein.

### The three peaks at 320–370 nm undergo different quenching by acrylamide

**Figure 3:**
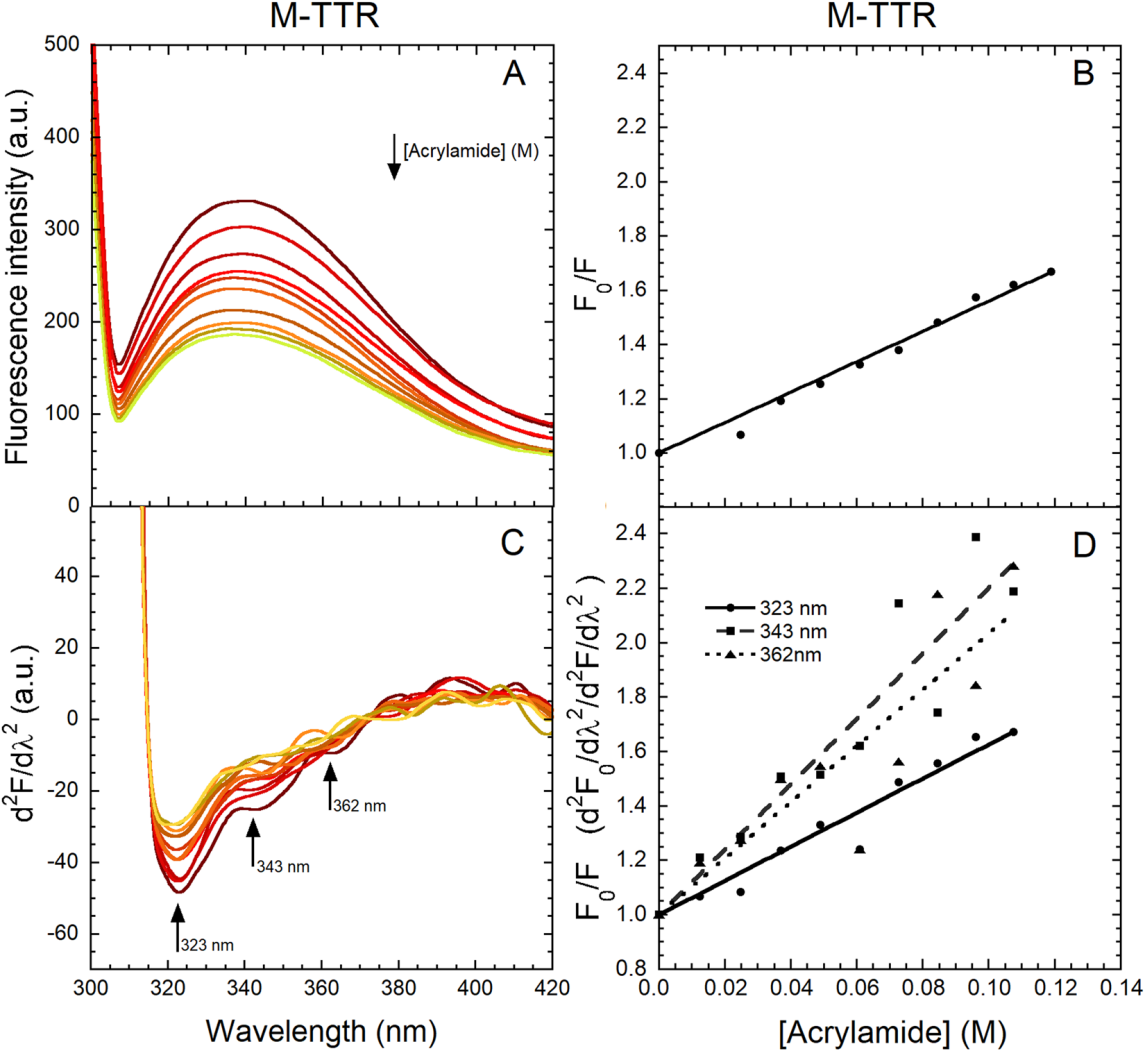
Quenching of M-TTR fluorescence spectra by acrylamide. (**A**) Fluorescence spectra (excitation 290 nm, slits 2.5 and 18 nm for ex and em, respectively) of 2.2 µM M-TTR following progressive additions of small volumes of 2.5 M acrylamide in 20 mM phosphate buffer, pH 7.4, 25 °C. (**B**) Plot of the ratio of total fluorescence intensity in the absence (*F*_0_) and presence (*F*) of acrylamide (*F*_0_/*F*) *versus* acrylamide concentration. The straight line represents the best fit of the data points to the Stern-Volmer linear function (Eq. 1) to determine the *K*_SV_ value. (**C**) Corresponding second derivative spectra with arrows indicating the major peaks at 323, 343 and 362 nm. (**D**) *F*_0_/*F versus* acrylamide concentration for the three indicated second derivative peaks, where *F*_0_ and *F* values are second derivative values. The lines through the data represents the best fit to the Stern-Volmer linear function (Eq. 1).

**Figure 4:**
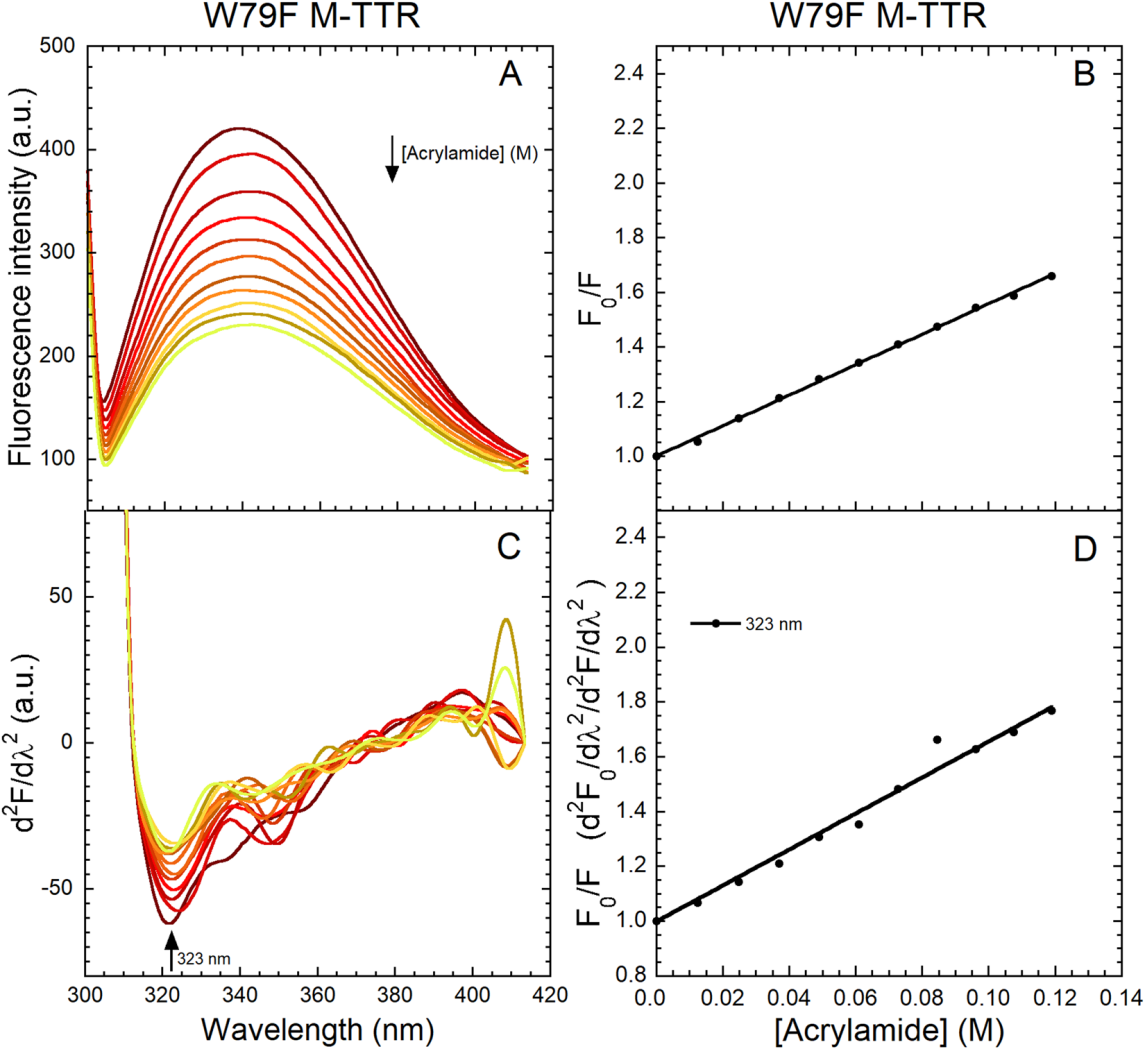
Quenching of W79F M-TTR fluorescence spectra by acrylamide. (**A**) Fluorescence spectra (excitation 290 nm, slits 2.5 and 14 nm for ex and em, respectively) of 3.5 µM W79F M-TTR following progressive additions of 2.5 M acrylamide in 20 mM phosphate buffer, pH 7.4, 25 °C. (**B**) Plot of the ratio of total fluorescence intensity in the absence (*F*_0_) and presence (*F*) of acrylamide (*F*_0_/*F*) *versus* acrylamide concentration. The straight line represents the best fit of the data points to the Stern-Volmer linear function (Eq. 1) to determine the *K*_SV_ value. (**C**) Corresponding second derivative spectra with the indicated major peak at 323 nm. (**D**) *F*_0_/*F versus* acrylamide concentration for the indicated second derivative peak, where *F*_0_ and *F* values are second derivative values. The lines through the data represents the best fit to the Stern-Volmer linear function (Eq. 1).

In order to investigate further the solvent-exposure of the three Trp41 conformers that give rise to the different peaks, we recorded the fluorescence emission spectra (290 nm excitation) of M-TTR and its W79F mutant in 20 mM phosphate buffer, pH 7.4, 25 °C, using progressive additions of acrylamide as a quencher of tryptophan fluorescence (Figs 3A and 4A). For these spectra, larger slit widths of emission were used to increase the fluorescence intensity at high acrylamide concentrations, where acrylamide is expected to quench the signal. The subsequent spillover of signal at low wavelength values (<306 nm) is indeed due to the excitation light that reached the detector by diffusion at these wavelength values. As expected, the increase of acrylamide concentration decreases the intrinsic protein fluorescence as the fluorescence of Trp41 is progressively quenched upon addition of acrylamide regardless of its adopted conformer and solvent exposure^42^. We plotted the ratio of total fluorescence intensity in the absence (*F*_0_) and presence (*F*) of acrylamide (*F*_0_/*F*) *versus* acrylamide concentration to determine the Stern-Volmer constant (*K*_*SV*_) value (see *Materials and methods* for details). These values were found to be 5.60 ± 0.13 M^−1^ for M-TTR and 5.58 ± 0.04 M^−1^ for the W79F mutant (Figs 3B and 4B). This reveals similar quenching degrees of the Trp41 residue for both variants.

We then converted all the spectra into their second derivative (Figs 3C and 4C) and produced plots of *F*_0_/*F versus* acrylamide concentration for the three identified second derivative negative peaks (Figs 3D and 4D). All three peaks decrease in intensity with the increase of acrylamide concentration, confirming that they arise from a tryptophan residue, i.e. Trp41. The two peaks at 343 and 362 nm decrease in intensity to similar degrees with acrylamide quenching and more effectively than the peak at 323 nm, suggesting that they arise from conformers that are more exposed to the solvent. The *K*_*SV*_ values for the three second derivative peaks of M-TTR were found to be 6.26 ± 0.28 M^−1^ at 323 nm, 12.01 ± 0.79 M^−1^ at 343 nm and 10.33 ± 0.97 M^−1^ at 362 nm. The analysis for the W79F mutant was not entirely successful as the high signal-to-noise ratio prevented from acquiring good second derivative spectra at the two higher wavelengths with consequently noisy Stern-Volmer plots. We could determine only a *K*_*SV*_ value for the 323 nm peak, which was found to be 6.57 ± 0.17 M^−1^, in agreement with that determined for the non-mutated M-TTR. Overall, we noticed a low *K*_*SV*_ value at 323 nm in both variants and this indicates a lower solvent-exposure of the corresponding Trp41 conformation relative to the other conformers.

### The second derivative spectra change with urea titration

**Figure 5:**
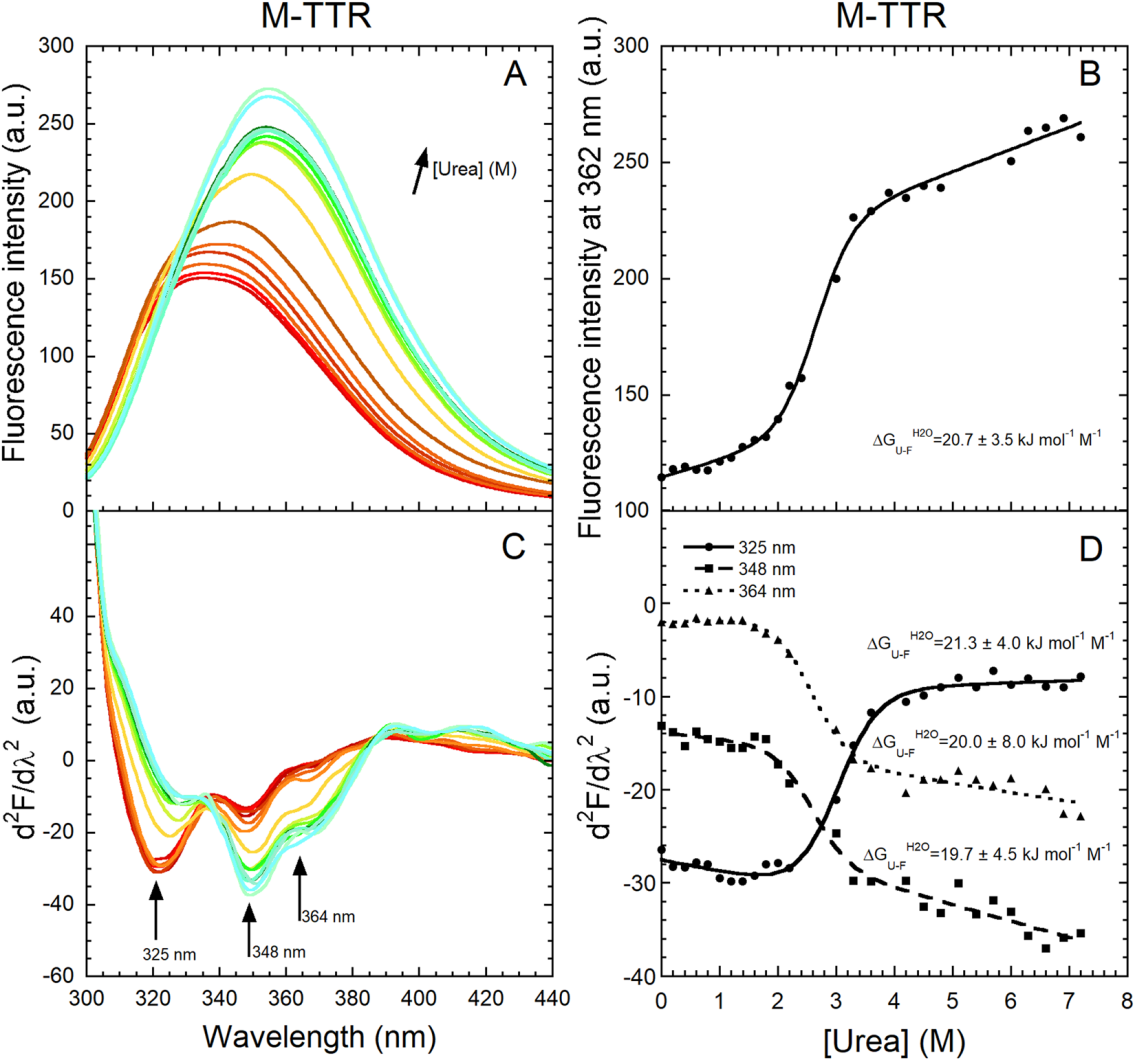
Equilibrium urea unfolding of M-TTR. (**A**) Fluorescence spectra (excitation 290 nm, slits 5 and 10 nm for ex and em, respectively) at 60 µM protein concentration and urea concentrations ranging from 0 to 7.2 M in 20 mM phosphate buffer, at pH 7.4, 25 °C. (**B**) Urea denaturation curve using fluorescence intensity at 362 nm. The solid line through the data represents the best fit to a two-state model (Santoro and Bolen, 1988). The obtained ${\rm{\Delta }}{G}_{U-F}^{H20}$ value is shown. (**C**) Corresponding second derivative spectra with arrows indicating the major peaks. (**D**) Values of second derivative at 325, 348 and 364 nm *versus* urea concentration, plotted to obtain urea denaturation curves. The solid lines through the data represent the best fit to a two-state model (Santoro and Bolen, 1988). The obtained ${\rm{\Delta }}{G}_{U-F}^{H20}$ values are shown.

We then acquired the fluorescence spectra (290 nm excitation) of M-TTR at various urea concentrations ranging from 0 to 7.2 M, again in 20 mM phosphate buffer, pH 7.4, 25 °C (Fig. 5A) and converted them into their second derivative (Fig. 5B). The gross spectra undergo a red shift of its wavelength of maximum fluorescence emission (λ_max_) and an increase of fluorescence emission intensity as the urea concentration increases (Fig. 5A). The first phenomenon occurs because Trp41 becomes more exposed to the solvent upon unfolding^32,34^, whereas the increase in fluorescence intensity is a probable consequence of the removal of Trp79 quenching as the protein unfolds^19,26^.

The plot of fluorescence intensity at the representative wavelength of 362 nm against urea concentration, i.e. the equilibrium urea denaturation curve, reveals a single cooperative transition between ca. 2.0 and 3.6 M urea and pre- and post-transition values that increase gradually and linearly with urea concentration, as a result of the lower quenching of tryptophan fluorescence by urea relative to water (Fig. 5B). The urea denaturation curve was analysed with a two-state unfolding model using a well-established procedure^43,44^, which provided quantitative measurements of the free energy change upon unfolding in the absence of denaturant (${\rm{\Delta }}{G}_{U-F}^{H20}$), its dependence on urea concentration (*m* value) and the concentration of 50% denaturation (*C*_*m*_). These values were found to be 20.7 ± 3.5 kJ mol^−1^, 7.8 ± 1.3 kJ mol^−1^ M^−1^ and 2.8 ± 0.2 M, respectively, in agreement with those previously reported of 20.4 ± 1.5 kJ mol^−1^, 6.9 ± 0.5 kJ mol^−1^ M^−1^ and 3.0 ± 0.1 M^26^.

In the second derivative spectra three peaks were again apparent in the region of light of 320–370 nm (Fig. 5C). The second derivative values at 325 nm, 348 nm and 364 nm were plotted *versus* urea concentration to obtain urea denaturation curves using the second derivative values rather than the gross fluorescence intensity values, as generally done (Fig. 5D). A first interesting observation is that the first peak at 325 nm decreases in intensity (becomes less negative) with urea concentration, whereas the second and third peaks at 348 and 364 nm increase (Fig. 5D). This indicates that the distribution of the three conformers of Trp41 observed in the absence of urea changes upon unfolding at high urea concentration. In particular, the population of the first conformer decreases and those of the other two increase upon unfolding (Fig. 5D). Such changes are cooperative, however, and occur within the same range of urea concentration. Indeed, the (${\rm{\Delta }}{G}_{U-F}^{H20}$), *m* and *C*_*m*_ values determined from such curves are similar, within experimental error, to each other and to those obtained here and previously^26^ from the fluorescence intensity values (Table 1).

**Table 1:** Thermodynamic parameters of M-TTR and W79F M-TTR urea-induced unfolding measured with conventional fluorescence and second derivative spectra^a^.

Parameter	Gross fluorescence at 362 nm	d^2^F/dx^2^ at 325 nm	d^2^F/dx^2^ at 348 nm	d^2^F/dx^2^ at 364 nm	Gross fluorescence (ref.^26^)
**M-TTR**					
${\rm{\Delta }}{G}_{U-F}^{H20}$ (kJ mol^−1^)	20.7 ± 3.5	21.3 ± 4.0	20.0 ± 8.0	19.7 ± 4.5	20.4 ± 1.5
m (kJ mol^−1^ M^−1^)	7.8 ± 1.3	7.1 ± 1.3	7.8 ± 2.9	7.7 ± 1.6	6.9 ± 0.5
C_m_ (M)	2.8 ± 0.2	3.0 ± 0.3	2.6 ± 0.3	2.5 ± 0.4	3.0 ± 0.1
**W79F M-TTR**					
**Parameter**	**Gross fluorescence at 330** **nm**	**d** ^**2**^ **F/dx** ^**2**^ **at** **324** **nm**	**d** ^**2**^ **F/dx** ^**2**^ **at** **346** **nm**	**d** ^**2**^ **F/dx** ^**2**^ **at** **367** **nm**	**Gross fluorescence** (**ref**.^26^)
${\rm{\Delta }}{G}_{U-F}^{H20}$ (kJ mol^−1^)	12.9 ± 1.3	12.9 ± 2.8	n.d	21 ± 14	13.0 ± 1.4
m (kJ mol^−1^ M^−1^)	4.2 ± 0.4	4.4 ± 0.08	n.d	6.6 ± 4.3	4.3 ± 0.4
C_m_ (M)	3.1 ± 0.2	3.0 ± 0.1	n.d	3.2 ± 0.2	3.0 ± 0.1

**Figure 6:**
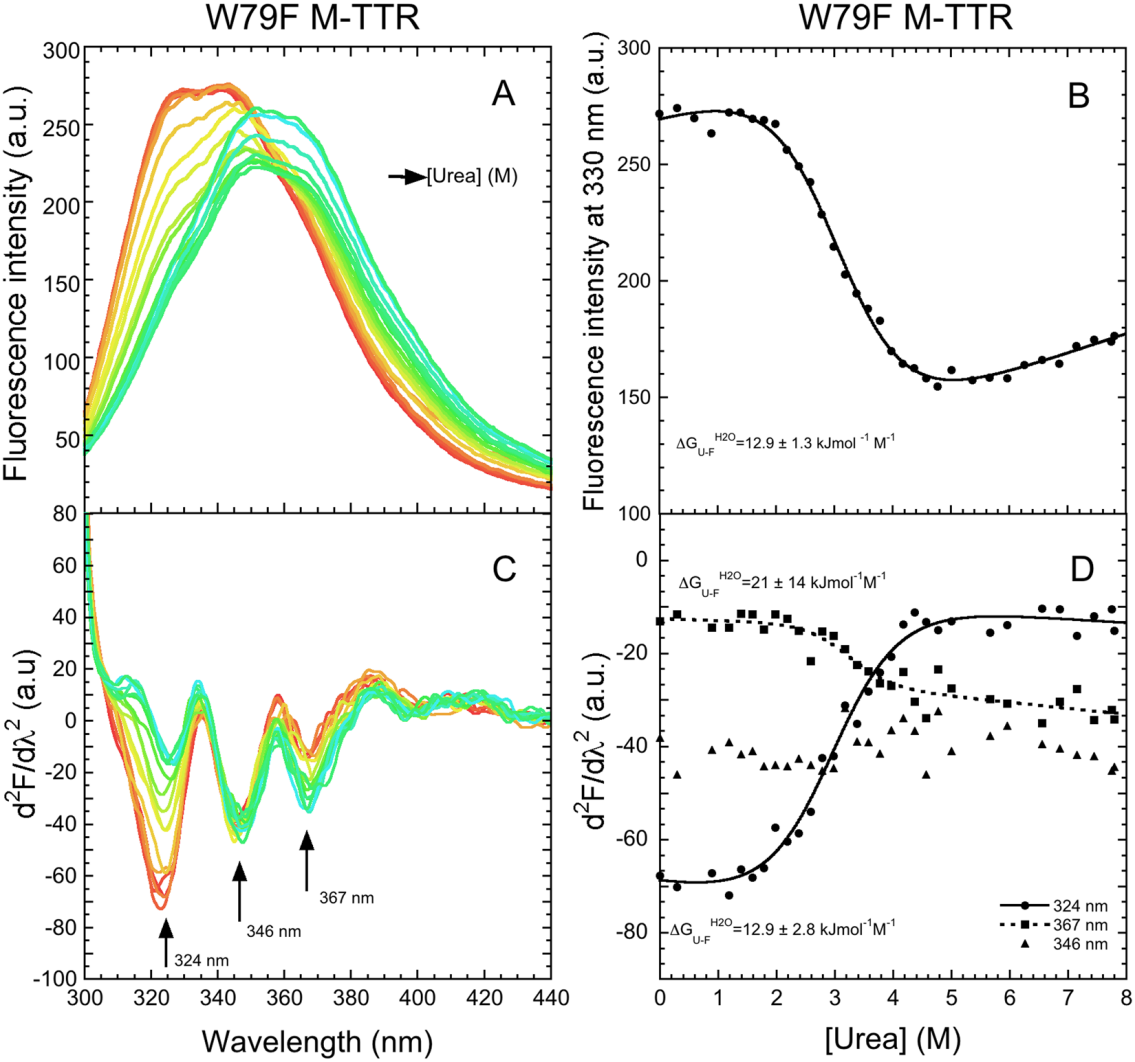
Equilibrium urea unfolding of W79F M-TTR. (**A**) Fluorescence spectra at 3 µM protein concentration and urea concentration ranging from 0 to 7.8 M in 20 mM phosphate buffer at pH 7.4, 25 °C (excitation 290 nm, slits 5 and 10 nm for ex and em, respectively). (**B**) Urea denaturation curve using W79F M-TTR fluorescence at 330 nm. The solid line through the data represents the best fit to a two-state model (Santoro and Bolen, 1988). The obtained ${\rm{\Delta }}{G}_{U-F}^{H20}$ value is shown. (**C**) Corresponding second derivative spectra with arrows indicating the three major peaks. (**D**) Values of second derivative at 324, 346 and 367 nm *versus* urea concentration, plotted to obtain urea denaturation curves. The solid lines through the data represent the best fit to a two-state model (Santoro and Bolen, 1988). The obtained ${\rm{\Delta }}{G}_{U-F}^{H20}$ values are shown.

All data are derived from the data shown in Figs 5 and 6, except those of the last column, previously determined^26^.

The analysis was repeated for the W79F mutant of M-TTR (Fig. 6). In this case the gross spectra have a red shift of the λ_max_ value, as observed for M-TTR, but no increase of fluorescence emission as the urea concentration increases, most probably because Trp79 is absent altogether and does not contribute to the overall fluorescence in the unfolded protein (Fig. 6A). The equilibrium urea denaturation curve at 330 nm reveals a single cooperative transition with ${\rm{\Delta }}{G}_{U-F}^{H20}$, *m* and *C*_*m*_ values of 12.9 ± 1.3 kJ mol^−1^, 4.2 ± 0.4 kJ mol^−1^ M^−1^ and 3.1 ± 0.2 M, respectively (Fig. 6B and Table 1), in agreement with those previously reported of 13.0 ± 1.4 kJ mol^−1^, 4.3 ± 0.4 kJ mol^−1^ M^−1^ and 3.0 ± 0.1 M^26^. In the second derivative spectra the three peaks associated with Trp41 at 320–370 nm are well defined (Fig. 6C). The first peak at 324 nm decreases and the third peak at 367 nm increases with urea concentration, whereas the second peak at 346 nm remains apparently stable (Fig. 5D). This indicates that the population of the first conformer of Trp41 decreases and that of the third conformer increases upon unfolding (Fig. 6D). Such changes are again cooperative, however, with ${\rm{\Delta }}{G}_{U-F}^{H20}$, *m* and *C*_*m*_ values similar, within experimental error, to each other and to those obtained from the gross fluorescence intensity values at 330 nm (Table 1). We should note, however, that the 367 nm transition is very subtle and the thermodynamic parameters have inevitably high experimental errors and that a transition with the 346 nm peak was not apparent altogether (Table 1).

### The second derivative spectra change with pH titration, but only at pH extremes

**Figure 7:**
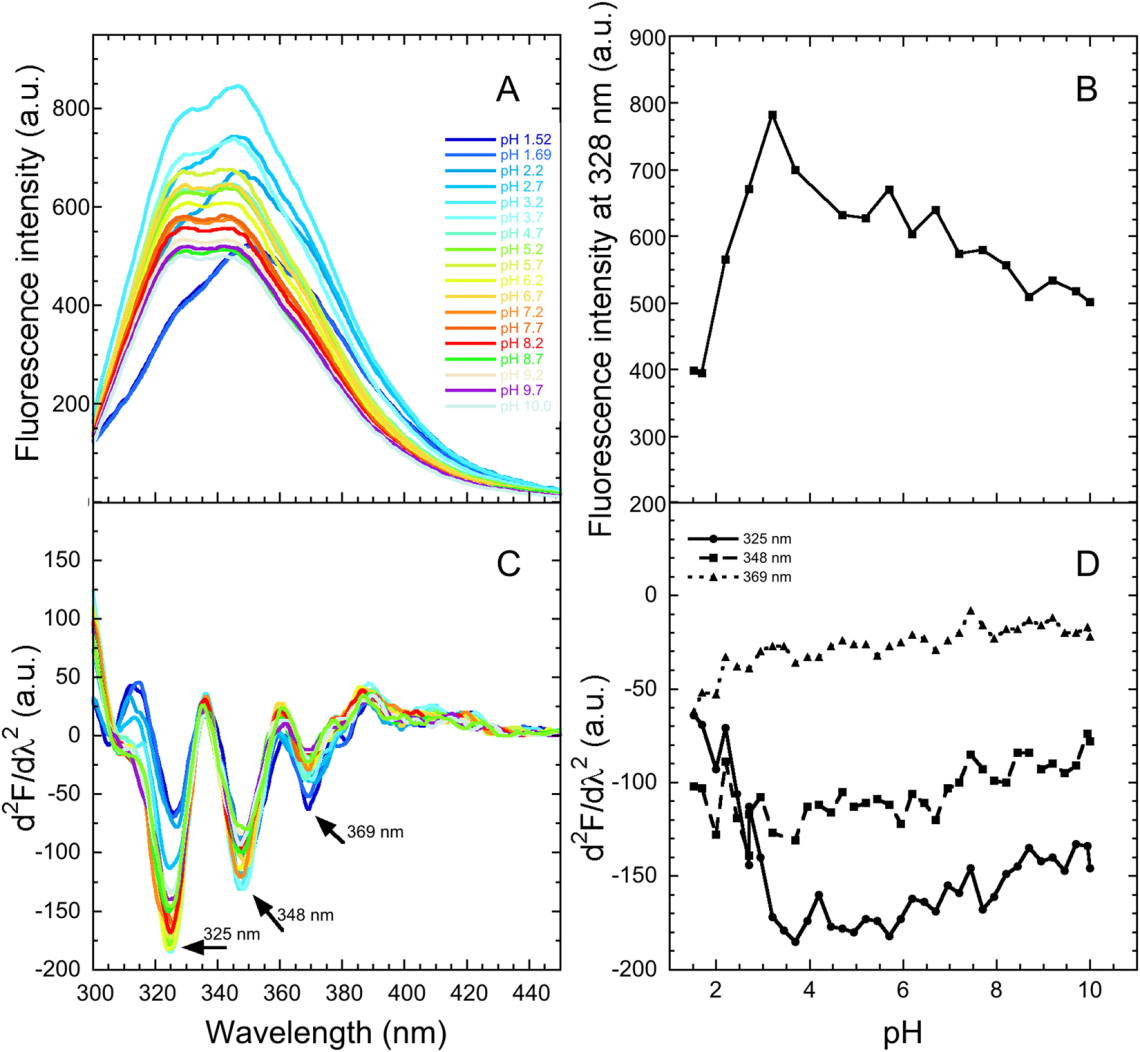
pH titration of M-TTR. (**A**) Fluorescence spectra (excitation 280 nm, slits 5 and 7 nm for ex and em, respectively) of 3 µM M-TTR in different buffers with the indicated pH values ranging from 1.52 to 10.0, total ionic strength 30 mM, at 25 °C. (**B**) Plot of fluorescence intensity value at 335 nm *versus* pH. (**C**) Corresponding second derivative spectra with arrows indicating the three major peaks. (**D**) Values of second derivative at 325, 348 and 369 nm *versus* pH.

We also extended our analysis at various pH values ranging from 10.0 to 1.5 to detect possible changes involving the Trp41 as the pH decreases from weakly alkaline to neutral values and then from neutral to acidic values. This investigated range of pH includes the window of pH 3.9–5.0, in which the amyloidogenic state of the protein is populated^19,20,24,26^. We therefore acquired the fluorescence spectra of M-TTR (excitation 280 nm) in 36 different buffers having different pH values, all having a constant ionic strength of 30 mM, a temperature of 25 °C and a protein concentration of 3 µM. The underivatized fluorescence emission intensity appears to increase linearly as the pH decreases from 10.0 to 3.4, with the spectra maintaining a similar overall shape (Fig. 7A). This is confirmed by the pH titration of the fluorescence intensity at a representative wavelength of 335 nM (Fig. 7B). Such a weak and linear behaviour is a typical solvent effect and rules out a significant change of the chemical environment of Trp41 within this range of pH values. The overall fluorescence emission then decreases, and a marked spectral shape occurs, as the pH decreases further down to 1.5 (Fig. 7A,B), indicating a change of the chemical environment around Trp41 at these extreme pH values.

The second derivative spectra are characterised by negative peaks at 325, 348 and 369 nm (Fig. 7C), whose values increase linearly (or become more negative) as the pH decreases from 10.0 to 3.4 (Fig. 7D), confirming the results obtained with the gross spectra. However, the peak at 325 nm decreases in intensity, the peak at 369 nm increases and that at 348 nm remains apparently constant, as the pH decreases further from 3.4 to 1.5. This indicates that the distribution of the three conformers of Trp41 observed at mildly alkaline, neutral or mildly acidic pH values changes upon unfolding at low pH. Interestingly, the change observed in the urea-unfolded state is more pronounced relative to the acid-unfolded state (Compare Figs 5D and 7D), indicating a lower degree of conformational change in the latter case. It is remarkable to observe, however, that the distribution of the three conformers of Trp41 does not change within the window of pH values where the amyloidogenic state is populated (pH 3.9–5.0). Nor does the chemical environment around the residue appear to be modified.

**Figure 8:**
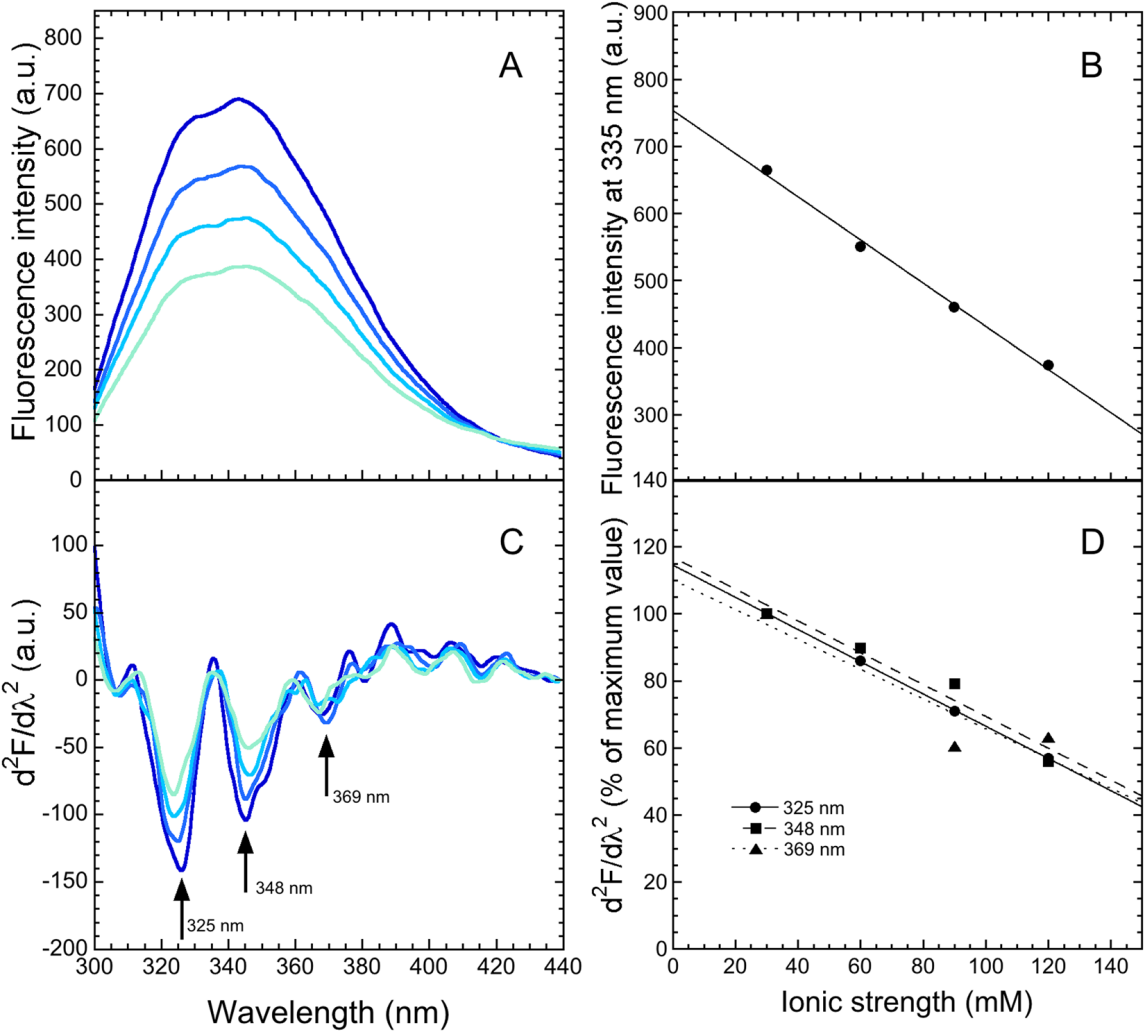
Gross and second derivative fluorescence spectra of M-TTR at low pH with various ionic strengths. (**A**) Fluorescence spectra of 125 µM M-TTR in 10 mM acetate buffer, pH 4.45, 37 °C at the indicated total ionic strength (µ) ranging from 30–120 mM (excitation 280 nm, slits 7.5 and 8 nm for ex and em, respectively). (**B**) Plot of fluorescence intensity value at 335 nm *versus* ionic strength. (**C**) Corresponding second derivative spectra with arrows indicating the three major peaks. (**D**) Values of second derivative at 325, 348 and 369 nm versus ionic strength. The lines through the data represent the best fits to a linear function of the form d^2^F/dλ^2^ = 100% + *a* · µ.

We also characterized the amyloidogenic conformation of M-TTR at pH 4.45 at various ionic strength ranging from 30 to 120 mM, as the increase of ionic strength is known to increase the rate of M-TTR aggregation^26^. We acquired the fluorescence spectra of M-TTR (excitation 280 nm) in 4 different samples having the same pH value of 4.45 but different ionic strength values due to progressive additions of a 2 M KCl solution up to 30, 60, 90, 120 mM ionic strength, after incubation at 37 °C for 30 min. The fluorescence emission intensity appears to decrease linearly with increasing ionic strength from 30 mM to 120 mM (Fig. 8A,B), suggesting a typical solvent effect, probably due to the fluorescence quenching by the chloride ion. The second derivative spectra are characterized by negative peaks at 325, 348 and 369 nm, which decreased in intensity (became less negative) to similar extents as the ionic strength increases, indicating that the observed distribution of Trp41 does not change (Fig. 8C,D). This finding suggests that the effect of the ionic strength on the aggregation rate of the protein at mildly acidic pH is due to changes in dynamics or conformation of protein regions other than Trp41 or to solvent effects, most likely arising from the ability of the salts to shield the electrostatic repulsions between individual M-TTR molecules.

## Discussion

The Trp41 residue of M-TTR is the only residue contributing significantly to the intrinsic fluorescence of M-TTR in a folded state, as Trp79 is quenched and manifests its fluorescence only in denatured states, in agreement with an unrelated analysis carried out on WT-TTR^19^. The fluorescence spectrum of Trp41 can be dissected in three main peaks that become apparent only in the second derivative spectra and correspond to three different conformers or rotamers of its side chain. Indeed, the three peaks have similar values of FRET efficiency with an acceptor located at Cys10, ruling out that one or more of such peaks arise from artifacts or other fluorophores present on the protein or in the sample as impurities.

The different wavelengths of maximum fluorescence of the three Trp41 conformers suggest that they have different degrees of hydrophobic contacts, hydrogen bonding and solvent exposure^32,45^. In particular, the first peak at ca. 322–325 nm can be assigned to class S tryptophan residues or, more likely, class I according to the classification previously proposed^32,45^: Class I residues are buried tryptophan residues with substantial hydrogen bonding (exciplexes with 1:2 stoichiometry), high relative polarity, low packing density and great microenvironment flexibility. The second peak at 342–344 nm can be assigned to class II or III: tryptophan residues with established contacts with structured (class II) or highly mobile (class III) water molecules and having total solvent accessibility and free dynamics. The third peak at 364–366 nm can only be a class III tryptophan. The results obtained by quenching the intrinsic fluorescence of M-TTR with progressive additions of acrylamide confirm the hypothesis that the 322–325 nm peak arises from a fairly buried conformer of Trp41, as the quenching is lower relative to the other two peaks, which appear by contrast to be fully solvent exposed and thus more susceptible to acrylamide quenching.

The distribution of the relative fractions of the three conformers change with urea-induced unfolding of M-TTR. The 322–325 nm fluorescence peak loses most of its intensity, whereas the other two peaks at 342–344 and 364–366 nm gain intensity as they are also enriched by the contribution from Trp79 that is no longer quenched and solvent exposed in the urea-denatured state. The urea-induced unfolding of the W79F mutant is more informative in this regard, as the fluorescence spectra of both the folded and unfolded states arise solely from Trp41. In this case we can observe a decrease and increase of the relative intensities of the 322–325 nm and 364–366 nm peaks, whereas the 342–344 nm peak remains constant. Overall, therefore, we have a conversion of some of the Trp41 moieties adopting the first conformation to the second and third conformation, and some of the Trp41 moieties adopting the second to the third. Interestingly, we also observed the maintenance of a residual intensity of the 322–325 nm peak in the urea-unfolded state, even at the highest urea concentrations tested here, well beyond the window of urea concentration where the main transition occurs. This indicates that a small fraction of the Trp41 residue maintains the most structured conformation adopted in the folded state and provides further evidence that residual native-like structure is retained, to a small but significant degree, in fully unfolded states of proteins^46,47^.

This scenario is similar following acidic-induced unfolding of M-TTR at pH extremes, with the first, second and third peaks decreasing, remaining constant and increasing in intensity, respectively. The unfolding is more modest in this case as the changes are all less marked relative to those observed in urea-induced unfolding of M-TTR (Compare Figs 5D and 7D). Again, the persistence of the 322–325 nm peak in the acidic-unfolded state indicates the presence of some degree of native-like residual structure in the Trp41 microenvironment.

The pH titration analysis, and the following ionic strength titration at the selected pH value of 4.45, also allow to determine that at the pH values of 3.9–5.0 and various values of ionic strength, in which the amyloidogenic state of the protein is populated^19,20,24,26^, the distribution of the three conformers of Trp41 is maintained with respect to that observed at neutral pH values and low ionic strength, where the protein is folded and fully soluble. This indicates that the microenvironment around Trp41 is maintained in the transition of M-TTR from the folded state to the amyloidogenic state. This finding is in agreement with other previously obtained observations. First, FRET measurements showed that the distance between Trp41 and Cys10 is maintained in the same range of pH values following this conformational conversion^26^. Second, solution nuclear magnetic resonance (NMR) studies revealed that the entire CBEF β-sheet, including the Trp41-containing β-strand C, maintains a fully native-like structure and the same dynamics following the transition of folded M-TTR and WT-TTR into the amyloidogenic state at moderately acidic pH values^22,24^. Indeed, a reconciling picture of the amyloidogenic state of TTR populated at moderately low pH that is in agreement with all data obtained so far is a largely native-like state with the D–E loop, the E–F α-helix (as well as its interconnected residues from the A–B loop), and the whole DAGH β-sheet exhibiting significant dynamical behaviour against a substantially packed CBEF sheet.

It will be interesting to extend these studies to include other techniques, such as near-UV CD and solution-state NMR in different conditions, to see if they can also detect a distribution of conformations for Trp41, and possibly other residues, and also determine their precise structure and how the distribution changes with solution conditions and mutations. The two solution NMR and X-ray structures deposited in the protein data bank (PDB entries 2NBO and 1GKO, respectively) have shown two different conformations for Trp41, as the spatial superimposition of the two structures reveals that the planes of the indole groups form an angle of *ca*. 90° due to a different rotation of the C_β_ − C_χ_ covalent bond, revealing that a structural plasticity exists for this residue. However, it will be interesting to detect such a conformational distribution within the same sample of M-TTR and how it changes in various contexts.

## Conclusions

In more generic terms, we have shown how the use of second derivative intrinsic fluorescence spectroscopy allows to determine the distribution of the various conformers of a given tryptophan residue in a given protein. It constitutes, therefore, a valuable probe to monitor the changes in conformation and dynamics of a protein of interest, such as those occurring following urea-induced unfolding, acidic-induced unfolding and the structural conversion into the amyloidogenic state for M-TTR. The various conformers can be followed individually during any given conformational conversion and the change of their distribution allows to determine not just the conformational change occurring for a give tryptophan residue and the change of its chemical microenvironment, but also the change of the microenvironment dynamics around it and the extent to which native-like conformers are maintained in a given non-native state.

## Experimental Procedures

### Materials

Dimethyl sulfoxide (DMSO), glutathione (GSH), Tris, citrate, formate, acetate, MES, phosphate, borate, trifluoroacetic acid (TFA), acrylamide, urea, N-acetyl-L-tryptophanamide (NATA) and L-tryptophan were purchased from Sigma-Aldrich (St Louis, MO, USA). *N*-(7-Dimethylamino-4-methylcoumarin-3-yl) maleimide (DACM) was purchased from Thermo Fisher Scientific (Waltham, MA, USA).

### Protein expression and purification

M-TTR and its W79F mutant were expressed and purified as formerly described^3^. They were then stored in 20 mM phosphate buffer, pH 7.4, −20 °C. In all cases, purity of the collected proteins was found by SDS-PAGE to be >95%.

### Protein labelling with DACM

Each protein variant was diluted to 0.2 mM in 20 mM phosphate buffer at pH 7.4, 25 °C. DACM aliquots solubilised in DMSO (99.9%) were added to a tenfold higher concentration of dye. The sample was covered with aluminium foil and incubated under shaking at 37 °C for 1 h. The reaction was stopped using 5 µl of TFA. The free dye was eliminated by dialysis (3.0 kDa molecular weight cut off), and then the sample was centrifuged. The concentration of DACM in labelled protein sample was estimated using *ε*_381_ = 27,000 M^−1^ cm^−1^, whereas protein concentration was calculated with *ε*_280_ = 18,450 M^−1^ cm^−1^ for labelled M-TTR and *ε*_280_ = 12,950 M^−1^ cm^−1^ for labelled W79F M-TTR. The contribution of an equimolar concentration of DACM-GSH at 280 nm was subtracted to calculate the difference. A normalization of absorbance spectra of DACM-GSH and DACM-M-TTR (or its mutant) was carried out.

### Fluorescence spectroscopy

A PerkinElmer LS 55 spectrofluorimeter (Waltham, MA, USA), provided with a thermostated cell holder attached to a HAAKE F8 water bath (Karlsruhe, Germany) and a 2 × 10 mm or 4 × 10 mm quartz cuvette were used for recording fluorescence spectra (290 nm excitation except pH titration and ionic strength analysis at 280 nm). For each sample three fluorescence spectra were acquired, blank subtracted and averaged. Intrinsic fluorescence emission spectra were transformed in their second derivative, which consists of the second order derivative of fluorescence (*F*) with respect to wavelength λ (d^2^*F*/d*λ*^2^). The second derivative spectra were calculated using the dedicated software for data analysis of the PerkinElmer LS 55 spectrofluorimeter and data windows of 30 points (15 nm).

### M-TTR, W79F M-TTR, NATA, L-tryptophan fluorescence spectra

0.5 ml of 3 μM M-TTR, or W79F M-TTR, or L-tryptophan or NATA in 20 mM phosphate buffer pH 7.4 were prepared to acquire fluorescence spectra from 300 to 460 nm at 25 °C in a 2 × 10 mm quartz cuvette (290 nm excitation). NATA is an N-terminal and C-terminal blocked analogue of L-tryptophan.

### FRET

For FRET measurements, unlabelled or DACM-labelled M-TTR (or its W79F mutant) were diluted to a concentration of 3 μM, in 20 mM phosphate buffer, pH 7.4, 25 °C and mixed to achieve different percentages of labelled and unlabelled M-TTR (or W79F M-TTR) from 0 to 100%. Fluorescence spectra were recorded from 290 to 450 nm (290 nm excitation) with a 10 × 2 quartz cuvette and the fluorimeter described above. The fluorescence emission spectrum of the unlabelled sample showed a peak at 330–350 nm and the labelled sample (100% DACM-M-TTR) a low intensity peak at 330–350 nm and an intense peak at 465 nm due to the energy transfer from excited tryptophan residues to the DACM group. Values of second derivative were plotted as a function of DACM-labelling percentage for the three peaks of M-TTR and W79F M-TTR in the window of 320–370 nm, after normalisation to attribute 100% to the second derivative values determined with 0% labelling.

### Fluorescence quenching by acrylamide

M-TTR variants were diluted to 2.2 µM (M-TTR) or 3.5 µM (W79F mutant) in 20 mM phosphate buffer, pH 7.4 to obtain samples of exactly 1000 µl and incubated at 25 °C. 5 µL aliquots of 2.5 M acrylamide solution (at 25 °C) were progressively added to 1 ml of protein sample up to 50 µL. Between the various additions, fluorescence spectra from 300 to 420 nm (290 nm excitation) were recorded under constant stirring using the fluorimeter described above and a quartz cuvette 4 × 10 mm. The plot of total tryptophan fluorescence *versus* acrylamide concentration was analysed using the following linear Stern-Volmer relationship:

1 $$\frac{{F}_{0}}{F}=(1+{K}_{SV}\,\ast \,[Acrylamide])$$

where *K*_SV_ is the Stern-Volmer constant and is proportional to the degree of tryptophan exposure to the solvent, *F*_0_ is the fluorescence spectrum area without acrylamide and *F* is that with acrylamide; all spectra are corrected by

2 $${F}={{F}}_{{0}}/{\rm{A}};\,{\rm{A}}={1000}/{1000}+{{V}}_{{\rm{a}}{\rm{c}}{\rm{r}}{\rm{y}}{\rm{l}}{\rm{a}}{\rm{m}}{\rm{i}}{\rm{d}}{\rm{e}}}$$

where *V*_*acrylamide*_ is the acrylamide volume.

The same analysis was repeated using the negative peak values of the second derivative spectra at 323, 343 and 362 nm of M-TTR and only at 323 nm of its W79F mutant, as the subsequent peaks (347 and 355 nm) where not considered for the noisiness of the spectra.

### Equilibrium urea unfolding

A number of samples of 60 µM M-TTR or 3 µM W79F M-TTR in 20 mM phosphate buffer, pH 7.4 with varying urea concentrations (0–7.8 M), were prepared and incubated at 25 °C for 1 h in a water bath. Fluorescence spectra were recorded at 25 °C from 300 to 500 nm (290 nm excitation) with a 4 × 10 mm quartz cuvette and the fluorimeter described above. The resulting plots of fluorescence intensity at 362 and 330 nm againts the concentration of urea were analysed with a two-state folding model^23,43,44^ for M-TTR and its W79F mutant, respectively, in order to obtain quantitative measurements of the free energy change upon unfolding in the absence of denaturant (${\rm{\Delta }}{G}_{U-F}^{H20}$), the concentration of middle unfolding (C_*m*_) and the dependence of the free energy change upon unfolding on urea concentration (*m* value). The same analysis was repeated using the negative peak values of the second derivative spectra at 325, 348 and 364 nm for M-TTR and at 324 and 367 nm for its W79F mutant.

### pH titration

36 samples of 3 µM M-TTR in 36 different buffers ranging from pH 1.52 to 10.0 and constant ionic strength of 30 mM were prepared and incubated in water bath at 25 °C for 30 min. The first three buffers ranging from pH 1.52 to 2.0 were prepared using HCl at 10, 20, 30 mM. In order to cover the remaining pH range 2.2–10.0, the buffers prepared were citrate (pH 2.2–2.9), formate (pH 2.9–3.9), acetate (pH 3.9–5.6), MES (pH 5.6–6.5), phosphate (pH 6.5–7.5), tris (pH 7.5–8.8) and borate (pH 8.8–10.0), all at 10 mM concentration and using NaCl to bring the total ionic strength to 30 mM. Fluorescence spectra were recorded at 25 °C from 300 to 450 nm (excitation 280 nm) using a 2 × 10 mm quartz cuvette and the fluorimeter described above. A plot of fluorescence intensity at 335 nm *versus* pH was obtained. Plots of negative peaks at 325, 348 and 369 nm of the second derivative spectra *versus* pH were also produced.

### Ionic strength analyses

4 samples of M-TTR at 125 µM in 10 mM acetate buffer, pH 4.45 that differed in ionic strength due to progressive additions of small volumes of a 2 M KCl solution up to 30, 60, 90 and 120 mM, were prepared and incubated at 37 °C for 30 min in a water bath. Between the various additions, fluorescence spectra from 300 to 440 nm (excitation 280 nm) were recorded using a 2 × 10 mm quartz cuvette. Corresponding second derivative spectra show negative peaks at 325, 348 and 369 nm.

## Data Availability

The datasets generated during and/or analysed during the current study are available from the corresponding author on reasonable request.
